# A Spatial Model of Mosquito Host-Seeking Behavior

**DOI:** 10.1371/journal.pcbi.1002500

**Published:** 2012-05-17

**Authors:** Bree Cummins, Ricardo Cortez, Ivo M. Foppa, Justin Walbeck, James M. Hyman

**Affiliations:** 1 Mathematics Department, Tulane University, New Orleans, Louisiana, United States of America; 2 Department of Epidemiology, Tulane University, New Orleans, Louisiana, United States of America; University of Michigan and Howard Hughes Med. Inst., United States of America

## Abstract

Mosquito host-seeking behavior and heterogeneity in host distribution are important factors in predicting the transmission dynamics of mosquito-borne infections such as dengue fever, malaria, chikungunya, and West Nile virus. We develop and analyze a new mathematical model to describe the effect of spatial heterogeneity on the contact rate between mosquito vectors and hosts. The model includes odor plumes generated by spatially distributed hosts, wind velocity, and mosquito behavior based on both the prevailing wind and the odor plume. On a spatial scale of meters and a time scale of minutes, we compare the effectiveness of different plume-finding and plume-tracking strategies that mosquitoes could use to locate a host. The results show that two different models of chemotaxis are capable of producing comparable results given appropriate parameter choices and that host finding is optimized by a strategy of flying across the wind until the odor plume is intercepted. We also assess the impact of changing the level of host aggregation on mosquito host-finding success near the end of the host-seeking flight. When clusters of hosts are more tightly associated on smaller patches, the odor plume is narrower and the biting rate per host is decreased. For two host groups of unequal number but equal spatial density, the biting rate per host is lower in the group with more individuals, indicative of an attack abatement effect of host aggregation. We discuss how this approach could assist parameter choices in compartmental models that do not explicitly model the spatial arrangement of individuals and how the model could address larger spatial scales and other probability models for mosquito behavior, such as Lévy distributions.

## Author Summary

Mosquito-borne diseases can spread when a mosquito bites a vertebrate host to obtain a blood meal for egg-laying. The first step in the transmission process consists of the mosquitoes seeking and finding a host. Mosquitoes use the wind direction and odors, such as carbon dioxide, emitted by the hosts in order to locate a host to bite. We present a spatial computational model of the host-seeking process in a region where hosts are heterogeneously distributed in clusters. The model is used to analyze the success in finding hosts once the mosquitoes are close to the host. We show that the number of mosquito-host contacts increases as hosts become more widely spaced within their clusters; that mosquito flight perpendicular to the wind leads to greater success in locating a host; and that the number of bites per host decreases when hosts aggregate into larger clusters.

## Introduction

The transmission of infectious agents is heterogeneous in the sense that the risk for infection and infectiousness are unevenly distributed over the population [1]. This heterogeneity is an important factor in predicting the spread of an infection; ignoring it may result in misleading inference about transmission dynamics by underestimating the probability that an infectious agent will persist [2]. Sources of heterogeneity in mosquito-borne disease transmission include uneven distribution of hosts and breeding sites [3], differences in host susceptibility to infection [4], and the nonuniform distribution of mosquito bites among spatially distributed hosts [5] (per-capita biting rates). The number of mosquitoes that bite a host depends, among other things, on the number that find it. Host finding by mosquitoes is largely driven by olfactory cues that are given off by individual hosts [6]. The spatial arrangement of hosts is likely to affect the spatial distribution of the odor plume and thus the mosquitoes' ability to locate and feed on them. For example, it may be easier for a mosquito to find a large roost of birds than to find an single bird. Unless the probability of finding a host is exactly proportional to the density of hosts, unevenly distributed contact rates on individual hosts, and thus heterogeneous disease transmission, will result. Understanding the dynamics of odor-driven mosquito-host interaction is fundamental to a detailed mechanistic understanding of mosquito-borne transmission.

Despite its important epidemiological implications, experimental data on the relationship between host aggregation and mosquito-host contact rates are sparse, and thus, little is known definitively about mosquito strategies for finding hosts. The goal of this work is to propose a modeling framework that can provide preliminary insights into the relative effectiveness of different host-seeking strategies used by mosquitoes. In this paper, we develop, assess, and utilize a mathematical model to simulate both the odor plume in the presence of wind and the host-seeking behavior of mosquitoes in response to that odor plume. Mosquitoes are modeled as discrete *agents* that fly continuously in search of discrete hosts (birds). The flight direction and speed of individual mosquitoes is influenced by wind and odors emitted by the hosts. The wind is composed of a deterministic, large-scale component plus a stochastic component to represent small-scale eddies and fluctuations. In this study we focus on mosquito host-seeking behavior within meters of a host, motivated by indoor experiments involving mosquito-bird interactions [7]. The Discussion section describes the potential for larger scale simulations that incorporate more of the host-seeking process. We hope that the model may serve as a virtual laboratory for testing different hypotheses for how mosquitoes use odor plumes to locate potential hosts and may offer guidance both to the planning of experimental studies and to the interpretation of experimental data.

This contribution joins a group of other important mathematical models for simulating the host-seeking dynamics of mosquitoes. Commonly, models are based on parameters such as contact rates and probabilities (of feeding, diversion, dying, etc.). Often these models assume homogeneous mixing and do not include space explicitly, so that the probabilities and contact rates are independent of location. In [8], the authors describe a kinetic, non-spatially explicit model to determine how much coverage is enough to protect individuals who do not use insecticide-treated nets (ITNs) in the context of malaria transmission. The model has also been modified to explore the effects of bed nets [9] and to introduce ‘bloodless’ hosts such as baited traps [10]. The latter also modeled the host-seeking process as a non-host oriented kinesis followed by a host-oriented taxis once the mosquito has encountered an odor cue. A related model for the mosquito feeding cycle addressing the effect of ITNs on malaria transmission is found in [11]. One model that does include space explicitly is that in [12] for plume finding in the context of an underwater substrate. The authors use a model in which the probability of detecting the plume depends on the seeker's movements, on the fluid motion, and on the plume shape in space and time. They report that as plume-detection success increases, the efficiency decreases (the process is slow). Our results are consistent with this observation.

### Mosquito host-seeking behavior

**Figure 1:**
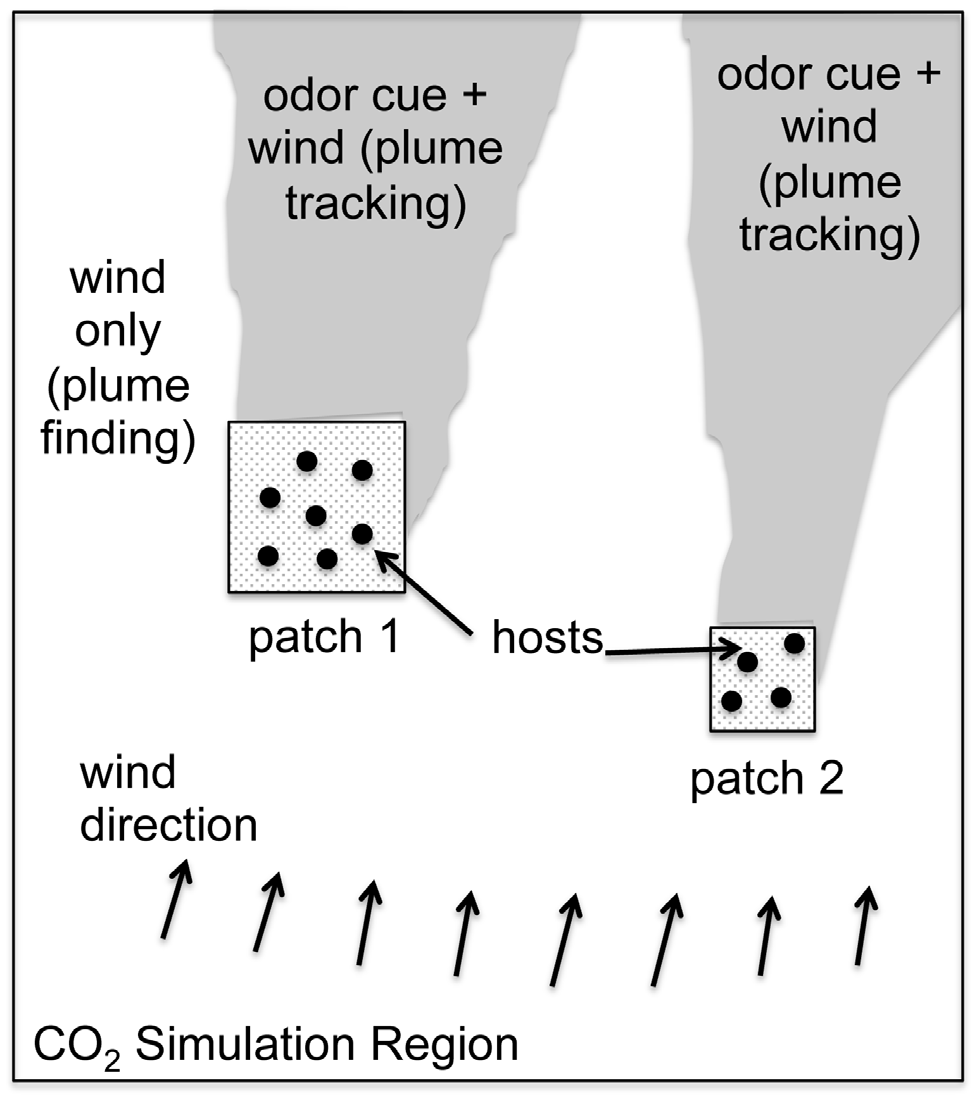
Computational domain. This is a schematic of the 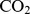 simulation region where the odor plume is computed. At the edges of the simulation region, the 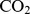 concentration is carried out by the wind. The patches represent smaller subregions where the hosts are located. The spatial distribution of hosts within each patch is uniform and there can be multiple patches. The hosts are stationary in the following simulations, although this is not a limitation of the model. The mosquitoes are initially placed in a subregion inside the 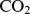 simulation region but are allowed to leave it (and possibly reenter it) during the simulation. The wind exists everywhere, even outside the 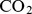 simulation region.

We provide a review of the biological literature that influenced our modeling choices. The scenario that we consider is illustrated in Fig. 1, where hosts are distributed in groups, or patches, and they emit an odor (e.g. 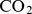) that gets carried by and diffused in the wind in the same way that a puff of smoke dissipates in time. Uniform, laminar wind extends host odor into a long, thin plume with sharp transverse gradients and shallow longitudinal gradients. If the wind is turbulent, the odor plume is highly intermittent, but still retains relatively shallow average longitudinal gradients compared to the transverse gradients [13]. We class mosquito host-seeking behavior as either *plume finding*, which is flight in search of an odor plume, or *plume tracking*, which is flight within the odor plume (these terms are adopted from [12]).

#### Plume finding: Absence of odor cues

There is little consensus in the literature about mosquito host-seeking behavior in the absence of odor cues. If wind is absent, orientation may be determined by large visual features in the environment [14] or may be characterized as a directionally unbiased random walk, also called *kinesis* [10]. If wind is present, then mosquitoes may deliberately choose to fly upwind, downwind, or crosswind in search of a host, which are types of directional searching referred to as *anemotaxis* [15].

Each of the three anemotactic behaviors has been described as plausible based on either experimental or theoretical work. Mosquitoes typically fly upwind in laboratory wind tunnels even when there is no odor present [16], [17]. In wind tunnel experiments, a low velocity artificial wind is blown across an odor source down an enclosure toward a mosquito entrance. Mosquitoes are released into the tunnel and their flight path is videotaped. In control experiments where no odor is added to the wind, many mosquitoes fly upwind and some even locate the “source” – the wind entrance into the tunnel. However, a set of field experiments reported in [18] provided evidence for downwind flights in host-seeking *Mansonia* spp. In these experiments, a human subject was surrounded on the downwind side by a tall, hemispherical fence of radius 18 m, to exclude mosquitoes using an upwind search strategy. By comparison with controls, the authors concluded that some mosquitoes employ a downwind plume-finding strategy. In [19] it is argued using geometry that crosswind searching is the most effective when the odor plumes are long and thin. If the variability of the wind direction is greater than 30 degrees, then a mathematical argument shows that upwind or downwind searching is optimal [20].

Mosquito response to wind depends on the strength of the wind. A typical mosquito flight speed is 1 m/s [15], [17]. As reviewed in [21], mosquitoes fly faster than the wind speed when they are in the low velocity boundary layer that forms adjacent to the ground. If they ascend too far (especially during the daytime when turbulence is greater), they are swept up and transported passively for long distances. Some mosquitoes may have adapted to use this as a deliberate migration mechanism. However, most of the flights that mosquitoes make are short-range, appetitive flights seeking a blood meal or oviposition site near the mosquito's home territory. Appetitive flights are disrupted by sufficiently high wind speeds, although the wind speeds at which this happens vary by species and geographic region. Wind speeds as low as 0.8 m/s (3 km/h) have been reported to drastically reduce the number of mosquito host-seeking flights, but no reduction in mosquito flight was documented in other situations for speeds as high as 3–8 m/s (11–29 km/h).

#### Plume tracking: Presence of odor cues

When a mosquito encounters an odor plume, it uses the odor plume and the wind to guide its flight to locate the host. Odor cues are complex olfactory signals released from a host's skin and breath. Many compounds are known to excite the chemoreceptors of mosquitoes. 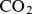, for example, is an important component in the odor plume that activates and helps maintain plume tracking [17], [22], [23]. Laboratory wind tunnel experiments indicate that sustained flight only occurs in the presence of an intermittent 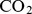 signal and not in uniform concentrations [23]. Other wind tunnel experiments suggested that broad, well-mixed 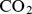 plumes may inhibit upwind flight while turbulent plumes of the same concentration induce upwind flight [16], [24], [25]. The importance of lactic acid, various aldehydes, and whole host odors for the location of hosts by mosquitoes has also been demonstrated [16], [22], [25], [26]. Dekker et al. [25] note that there is not a single model that fits mosquito flight response to all relevant host odors, even within a single species.

Mosquitoes exhibit different behavior in windy and windless conditions. In the absence of wind within the odor plume, mosquitoes must rely solely on odor cues [13] or on large features in the visual environment [14]. Mosquitoes probably estimate the direction of odor increase (the gradient) by the mechanism of *klinotaxis*, as is conjectured for tsetse flies [27]. During klinotaxis, an organism samples the host odor at one location, then moves and samples it again, using its memory of the concentration to choose the next direction [13]. Laboratory and field wind tunnel experiments suggest that under windy conditions within the odor plume, the mosquitoes travel upwind to locate the source [16], [24], [28]. Mosquitoes appear to infer wind direction from the optical flow of ground features relative to their position [27]. It is known that many organisms have characteristic turns in their upwind flight path (e.g. moths [13], [27]), but mosquitoes exhibit highly irregular upwind flight [29].

## Model

We formulate our model for the intermediate range flights of night-active mosquitoes such as *Culex quinquefasciatus* feeding on roosting birds, incorporating the biological information from the previous section. We construct a model that accommodates different plume-finding and plume-tracking mosquito behaviors, wind velocities and host locations. The model is two-dimensional and all of the dynamics are assumed to take place at a fixed distance from the ground.

### Odor plumes

**Figure 2:**
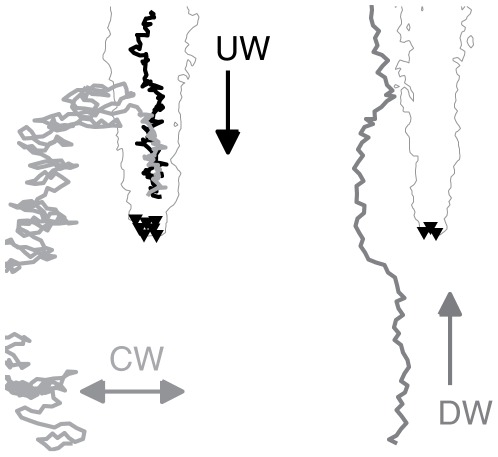
Examples of mosquito trajectories. Example mosquito trajectories for upwind (UW), downwind (DW), and crosswind (CW) plume-finding behaviors. The stationary hosts are distributed into two groups and the contour of the odor plume marks a snapshot of the 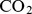 sensing threshold of the mosquito. The UW and CW mosquitoes successfully locate a host; the DW mosquito is unsuccessful. The CW mosquito leaves and re-enters the domain.

In the model, hosts emit a single gaseous compound that attracts mosquitoes, is convected by the wind, and diffuses in the air. For the purpose of this paper, we assume that the attractant is 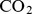. The 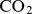 distribution over time is modeled by a convection-diffusion partial differential equation. The convection velocity of the wind is given by a vector 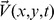, and the concentration 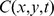 of 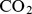 is described by the equation
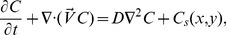
(1)where 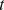 is time and 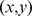 are spatial coordinates on a square domain of length 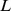. Throughout the paper, we refer to this square computational domain as the “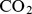 simulation region.” The constant diffusion coefficient 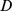 reflects the rate at which 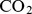 diffuses in air in the absence of wind. The term 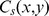 represents the odor cue emitted at a constant rate by the hosts in units of ppm/s:
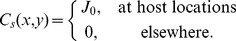
The wind velocity 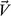 consists of two components:
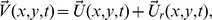
where 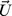 is used to introduce drifts or relatively large features produced by the air and 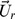 is a stochastic velocity vector used to approximate the effect of small-scale wind variations in the domain. The direction of 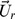 at each point in the domain is chosen uniformly from 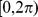 and the magnitude is chosen from a normal distribution centered around zero. The large-scale velocity field 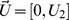 is a constant speed flow from bottom to top (see Fig. 2) in most of our simulations. However, we sometimes use a meandering plume 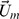 in place of the straight plume 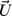, which is given by the expression
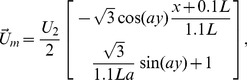
(2)where the frequency 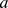 is 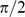. It is easily checked that this flow is incompressible.

Equation (1) is numerically evolved using second-order centered differences to approximate the Laplacian and a first-order conservative upwind finite difference method for the convection term (similar to page 636 of [30]). The normal components of the concentration gradient are zero at the boundary (Neumann conditions). We integrated the equations with a forward Euler method and confirmed that our solution converged by verifying that significantly decreasing the spatial grid size and time step size had a minimal change in the solution.

In most of the simulations in this paper, we consider length scales and time frames consistent with the mosquitoes being in close proximity to the hosts. The length of a side of the square domain 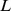 is 10 m and the simulations cover time periods of 50–500 seconds. The hosts are situated in the middle of the domain, 5 m from the top and bottom domain edges (see Fig. 2). Initially, the domain is bare of 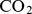 and it takes about 45 seconds for the plume to reach the domain edge. At that point, the mosquitoes are released into the domain, with starting positions dependent on their particular flight behavior. For upwind plume-finding behavior, the mosquitoes are all released downwind of the hosts; for downwind and crosswind behaviors, the entry is along the upwind side of the domain.

### Mosquito behavioral rules

Mosquitoes and hosts are modeled as discrete individuals, or agents. Hosts are stationary, motivated by the interaction between nocturnal *Cx. quinquefasciatus* mosquitoes and their roosting bird prey. In this section we explain the mosquito navigation model, which differs during plume finding and plume tracking. Assumptions about mosquito agent behavior include:

- The mosquitoes do not affect each other.
- The mosquito motion is not restricted to the 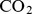 simulation region and may leave and re-enter the region during their random walks.
- Below a 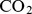 threshold 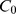, the mosquitoes navigate only using wind direction and move according to a predetermined upwind, downwind, or crosswind pattern. This is plume-finding behavior.
- For concentrations above the threshold 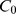, the mosquitoes respond by changing to plume-tracking behavior: moving upwind and toward larger levels of 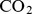. There is a saturation concentration level 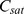 above which no further changes in concentration can be detected.
- Large concentration levels strongly bias the mosquito flight direction and result in flights that are on average closer to the target direction. Conversely, low concentrations weakly bias the mosquito flight direction and result in greater variability in flight direction.
- When a mosquito comes within a predetermined radius 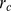 of a stationary host, the mosquito is removed from the simulation and a “contact” is recorded. In this context, a contact means an attack on the host, regardless of whether it results in a blood meal, a diversion, or death [10]. The term “contact rate” refers to the number of contacts per time period, usually the length of one simulation.
- Mosquito agents may move toward higher concentration levels by one of two mechanisms – temporally sampling the concentration level or directly sensing the spatial gradient of the concentration (*klinotaxis* and *tropotaxis* respectively in chemotaxis literature [13]).

#### Mosquito flight direction based on 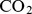 concentration (klinotaxis)

**Figure 3:**
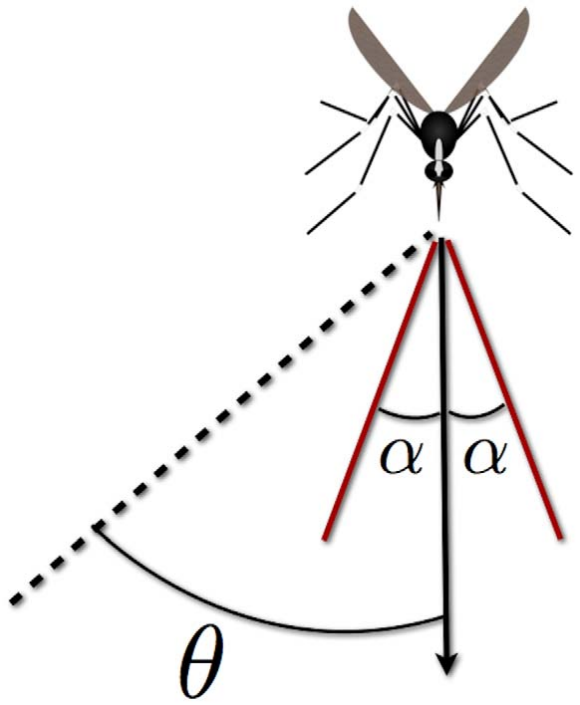
Mosquito flight direction choice. Schematic of the mosquito direction choice in the host-seeking model. At each discrete flight segment, the angle 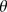 represents the ideal target direction of the mosquito, which can only be sensed or followed to a precision of 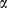.

During plume-tracking behavior, the mosquito chooses a flight segment direction by comparing the current 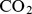 concentration level with the concentration previously encountered (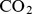 levels are interpolated to the mosquito location). If the 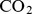 level is higher, the mosquito is biased to continue in the same direction. If it is lower, the mosquito is biased to turn around. So the target direction of the mosquito, 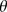, is either plus or minus its previous direction. Given that there is inaccuracy in the ability for a mosquito to sense 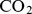 levels, we add stochasticity to the ultimate choice of direction by constructing a window of width 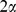 centered around 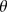, 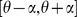 (see Fig. 3).

The quantity 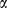 is dependent on the magnitude of the 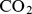 change. To calculate it, we set a maximum concentration change that can be sensed, calling it 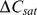, and a minimum concentration change 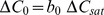, with 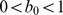. Concentration changes below 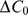 are imperceptible. We define a relative concentration change by 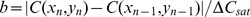, where 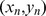 is the mosquito location at time 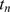. Then 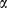 is computed from
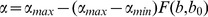
(3)where 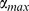 and 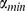 are the maximum and minimum allowable window half-widths and 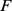 is a ramp function that takes values between 0 and 1:
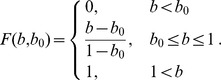



The mosquito direction influenced by the 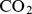 concentration, 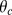, is chosen randomly from a uniform distribution in the window interval:
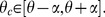
(4)The interval size is largest when the sensory input is lowest, leading to greater uncertainty in direction choice. We refer to this rule as the “sampling method” in the remainder of the paper.

#### Mosquito flight direction based on 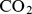 gradient (tropotaxis)

Gradient chemotaxis models use the concentration gradient, 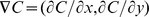, as the variable sensed by mosquitoes or other chemotactic agents [31], [32]. This model is incorporated into our framework by computing the 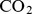 gradient on the grid and interpolating it to the mosquito location. The direction of the gradient is the target direction 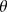 for the mosquito's next flight segment. Following the procedure in the previous section, we set 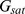 as the maximum and 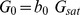 as the minimum gradients that can be sensed, and define the relative gradient at the mosquito location by 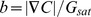. Mosquito direction is then computed as in Eqs. (3)–(4). We refer to this as the “gradient method.”

#### Mosquito flight direction based on wind velocity

Mosquito agents have a preassigned plume-finding strategy: downwind, upwind, or crosswind. The target angle for wind, denoted 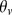, depends on the wind direction at the mosquito location, which is evaluated from the large scale wind 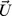 plus a random component interpolated from the grid in the 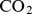 simulation region. For example, if wind is blowing from South to North, a mosquito with a downwind strategy would have a target angle pointing North; a mosquito with an upwind strategy would have a target angle pointing South; and one with a crosswind strategy would have a target angle pointing either East or West (Fig. 2).

The size of the angle window is chosen according to the strength of the wind 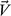 sensed by the mosquito. We assume that mosquitoes can distinguish wind speeds up to a saturation value 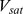. The direction is chosen randomly from a precision window
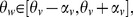
(5)where 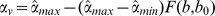 with 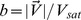 similar to Eq. (3). The threshold speed for mosquito response to wind is a percentage of the saturation value, 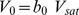. During crosswind plume-finding behavior, the duration of travel in one direction is a parameter in the model. We choose the duration to be uniformly selected from an interval 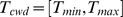.

#### Mosquito flight speed

A mosquito agent chooses a flight speed 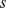 between a minimum 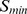 and a maximum 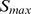 depending on the local 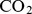 level. Unlike the random choice of direction, the speed of the mosquito is defined by a deterministic formula similar to Eq. (3):
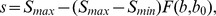
where the variable 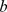 represents the scaled 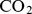 concentration 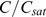 at the mosquito location. If the concentration is below the threshold 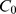, then the mosquito speed is 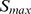. Similarly, if the concentration is above 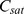, then the segment speed is 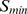. This allows mosquitoes to remain near areas of high 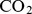 concentration. A change in speed due to the presence of a chemical signal is properly called *orthokinesis* [33].

#### Mosquito position updating

The direction of the flight segment and the speed of each mosquito are updated every 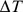 time units. The updated mosquito position is calculated by
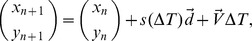
where 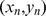 is the mosquito position at time step 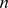. The last term is passive convection of the mosquito in the ambient wind. The quantity 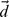 is a direction vector that varies between plume finding and plume tracking:
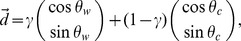
where 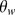 from Eq. (5) is based on the wind and 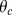 from Eq. (4) is based on the 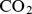. In plume finding, when there are no odor cues, 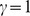, and the flight direction depends only on the assigned plume-finding behavior of the mosquito. During plume tracking, 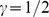 and the flight direction is the average of the directions chosen from the concentration and from an *upwind strategy*. Mosquitoes that have a downwind or crosswind plume-finding behavior will begin to fly upwind in the presence of 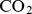.

### Assessment of the model

We assess model performance by comparing different mosquito navigation strategies and by evaluating the sensitivity of the model to a subset of the simulation parameters. We find that the gradient and sampling methods can exhibit comparable performance and that, in general, the parameter set in Table 1 is robust to small changes.

**Table 1:** Parameter choices.

Parameter	Value	Description
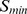	0.4 m/s*	minimum mosquito flight speed
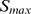	1.5 m/s*	maximum mosquito flight speed
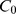	40 ppm*	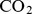 sensing threshold (no ambient 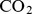 )
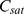	4000 ppm	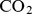 sensing saturation (no ambient 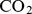 )
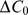	0.2 ppm	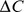 sensing threshold
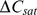	80 ppm	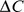 sensing saturation
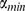	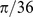 *	minimum 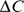 interval half-width
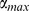	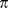	maximum 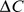 interval half-width
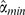	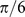 *	minimum wind-sensing interval half-width
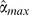	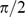	maximum wind-sensing interval half-width
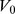	0 m/s	wind-sensing threshold
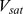	0.5 m/s	wind-sensing saturation
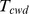	[0.5, 0.9] s*	duration of crosswind flight
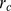	0.5 m*	critical radius for mosquito-host contact
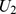	0.2 m/s	speed of large-scale wind
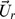	[X(t), Y(t)]0.15 m/s*	superposed stochastic small-scale wind velocity
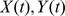	mean 0, std dev 0.5	Gaussian random variables resampled every 2 s
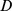	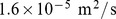 *	diffusion coefficient of 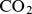 in air
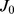	1680 ppm/s*	rate of 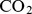 release from a host
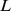	10 m	length of a side of the square domain
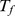	up to 500 s	simulation time length
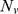	200	number of mosquitoes per simulation

Parameter symbol, value, and description for the simulations in the Methods and Results sections. The first 14 parameters are related to mosquito navigation. The remainder control the simulated wind, 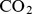 spread, and size of the simulations.

*: Starred values were locally varied in the sensitivity analysis.

#### Comparison of simulated host-seeking mechanisms

One might expect that perfect knowledge of the gradient would result in better performance than perfect knowledge of the 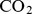 concentration. However, the model includes a window around the gradient direction from which the direction of motion is chosen randomly; in other words, the knowledge of the gradient is imperfect. We find that sufficiently imperfect knowledge of the gradient direction can result in overall behavior that is comparable to the sampling method (with different parameter values). This is an important observation since many existing models use the popular Keller-Segel approach that assumes knowledge of the concentration gradient [31], [32].

We placed nine hosts in a regular square grid at 1 ft (0.3 m) intervals in the center of the simulation region in the presence of a meandering velocity field. We examined upwind, downwind, and crosswind plume-finding behaviors, each in combination with the gradient and sampling methods for 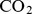 sensing. For the gradient method, we used the parameters 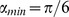, 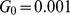 (40 ppm/meter), and 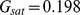 (7900 ppm/meter). The last two parameters are analogous to 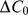 and 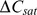 for the sampling method. See Eq. (2) for the formula governing the wind and Table 1 for all other simulation parameters. For convenience, all numerical simulations were computed using dimensionless variables, but we report parameters in dimensional units.

We also simulated a random walk strategy (with equal probability of stepping in all directions) in which the odor plume is not sensed at all and the wind has no effect. This mimics mosquito dispersal (mathematical diffusion) independent of the chemical concentration and wind. The comparison of the chemotactic rules to this random walk provides a baseline for determining how different two rules are. The random walk mosquitoes started at the same location as the downwind strategy, but chose a direction at every time step from a uniform distribution on 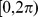. Their speed was constantly 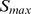, the same speed as the plume-finding behavior of the host-seeking mosquitoes. There are many other possible choices for random walks; see [12].

**Figure 4:**
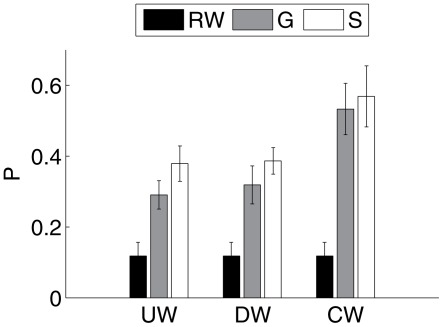
The proportion of mosquitoes finding a host within 150 s. The black bars are the results for a random walk (RW) without wind and 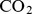, the gray for the gradient method (G) of concentration sensing, and the white for the sampling method (S) of concentration sensing. UW = upwind plume finding, DW = downwind plume finding, and CW = crosswind plume finding. The thin error bars are 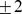 standard deviations. If the simulation is allowed to progress beyond 150 s, then no appreciable changes occur in the results for the host-seeking methods, but the random walk continues to accrue contacts.

The proportion (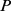) of mosquitoes that found a host after 150 s is shown in Fig. 4 for all the mosquito heuristics discussed above. The values shown in the bar graph are averages over 15 simulations in which the time series of random velocity fields is fixed, but the mosquito behavior stochastically varies. The thin error bars represent 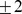 standard deviations. The random walk (black bars) had by far the fewest contacts, while the gradient and sampling methods have overlapping error bars. After 500 s, there was almost no change in either of the host-seeking methods, but the proportion of mosquitoes that found a host during the random walk increased to 0.25.

For these particular parameter choices, the gradient method and sampling method show similar results and both are substantially different than a random walk. There are enough free parameters in our model of host-seeking so that behavioral heuristics based on either spatial gradient sensing or temporal sampling can deliver approximately the same results. Since mosquitoes almost certainly navigate using klinotaxis [27], we will use the sampling method in the remainder of this work. However, we emphasize that our observations support the use of the Keller-Segel model of gradient sensing as in [31], [32].

#### Sensitivity analysis

The goal of this paper is to characterize the effect of host aggregation and mosquito plume-finding behavior on the mosquito-host contact rate for the parameters in Table 1. In this section, we consider a single host distribution and locally vary a subset of our model parameters independently to assess the robustness of the model with respect to our target parameter set. We find (with a few exceptions) that the parameter set we use with our model is robust to small changes in input parameters.

We simulated crosswind, downwind, and upwind plume-finding behaviors in the presence of a straight odor plume produced by an arrangement of nine hosts spaced 1 ft (0.3 m) in a square patch in the center of the domain. For each plume-finding behavior, we varied each of the ten starred parameters in Table 1 by 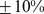 while holding all other parameters constant. To capture the average mosquito behavior, the simulations were repeated 15–20 times for each plume-finding behavior (upwind, downwind, and crosswind), until a mild convergence criterion was satisfied. In each set of simulations, we used the same sequence of random velocity fields and the same set of random numbers for mosquito behavior, so that the differences between sets were due solely to the parameter perturbations and not to the stochastic nature of the agents or wind. We analyzed two output variables:

- the proportion of mosquitoes that find a host anywhere in the domain, 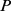, and
- the average time to locate a host, 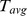.

When calculating 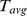, we exclude mosquitoes that never locate a host.

A local sensitivity index, 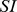, is a partial derivative of an output variable with respect to an input parameter that is scaled to allow comparisons across variables. High sensitivities are synonymous with large rates of change, while low sensitivities (and model robustness) are associated with small derivatives. The sign of 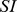 indicates if the change is in the same direction (positive) or the opposite direction (negative) as the perturbation. To calculate 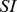 values, we used a centered difference approximation to the partial derivative multiplied by the baseline value of the input parameter over the baseline value of the output variable. Symbolically, if we let 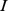 be an input parameter, we have
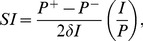
(6)where 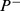,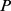, and 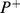 are the values of the output variable corresponding to the varying input parameter: 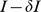, 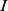, and 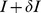, respectively. Since we consider variations of 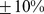, we choose 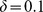. We estimated the error in this method by taking half of the difference between the one-sided partial derivatives:
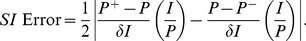
(7)


We show the combinations of plume-finding behavior, input parameter, and output variable with the highest sensitivity indices in Table 2. 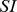
s that are small compared to 1 in absolute value indicate a robust response to parameter variation. In the first row, 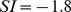 means that if the maximum speed of a mosquito (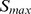) engaging in upwind plume finding increases by 10%, then the average time for a mosquito to find a host will be decreased by an estimated 18% with error bounds of 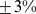. This is a large response and represents high sensitivity.

**Table 2:** Most sensitive local variation.

plume-finding behavior	output (baseline value)	input	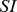	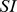 Error
upwind	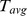 (11.6 s)	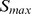	−1.8139	0.3155
upwind	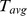 (11.6 s)	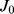	0.3035	0.1661
downwind	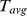 (4.6 s)	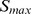	−0.6432	0.6093
downwind	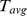 (4.6 s)	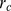	−1.0265	0.8221
downwind	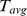 (4.6 s)	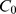	−0.6312	0.6741
downwind	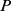 (0.20)	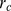	0.3156	0.0374
crosswind	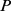 (0.36)	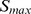	0.7292	0.2666

This table lists the highest sensitivity indices over all plume-finding behaviors and starred input parameters in Table 1 for the output variables 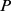 (proportion of mosquitoes that found a host) and 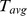 (the average time to a contact). The second column is the output variable with its baseline value at the parameter set given in Table 1. The third column lists the input parameters associated with the highest sensitivity indices. The fourth and fifth columns are the sensitivity index 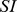 and its error from Eqs. (6)–(7). If 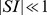, then the measured output *is not sensitive* to small variations in the input parameter. All combinations not shown have 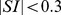.

In Table 2, there are two 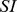
s very close to 0.3, and five greater than 0.6. The values greater than 0.6 represent moderate to high sensitivities, while the two values near 0.3 are reasonably low. All combinations that are not shown in Table 2 have 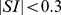. The total number of combinations tested were 20 per plume-finding behavior, or 60 total. We see low values of 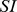 in most of the combinations tested, indicating that our model is locally robust to the parameter set in Table 1, although we do not characterize variability due to stochastic effects. The biggest exception to this robustness is the maximum flying speed of the mosquitoes (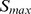), which strongly affects 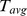 in upwind and downwind behaviors and moderately affects 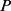 in crosswind plume-finding behavior. Overall, the average time to locate a host, 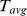, is more sensitive to input changes than the proportion of mosquitoes that find a host, 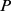.

## Results

We use the model introduced in the previous section to address several questions pertaining to space-dependent small-scale plume encounter and host localization.

- How do the different plume-finding behaviors compare in terms of effectiveness in finding a host?
- How does the number of contacts vary between two unequally-sized host groups?
- How does the number of contacts vary as the density of hosts changes in a small patch?

In the following subsections, we describe the simulations addressing these questions and we summarize our findings. We find that the most contacts occur when the mosquitoes use a crosswind strategy and are allowed sufficient time to either find a host or leave the domain permanently. We conclude that the larger of two unequally-sized groups has a smaller per capita number of contacts. Finally, we find that the number of contacts increases as hosts occupy a larger subregion within the computational domain.

### Assessing plume-finding behavior

Intuitively, a crosswind flight strategy should result in a larger number of contacts than upwind or downwind behavior if the plume is straight. This is because mosquitoes close to the plume are more likely to intercept it if their motion is primarily crosswind. But for a meandering plume, it is not obvious if a crosswind flight strategy is more effective than an upwind or downwind strategy. We simulated mosquito behavior in both straight and meandering plumes and recorded the proportion of mosquitoes that found a host and the average time that it took a mosquito to locate a host. We estimated the effectiveness of each plume-finding behavior using these results and found that a crosswind strategy is superior to upwind and downwind strategies, but is less efficient.

**Figure 5:**
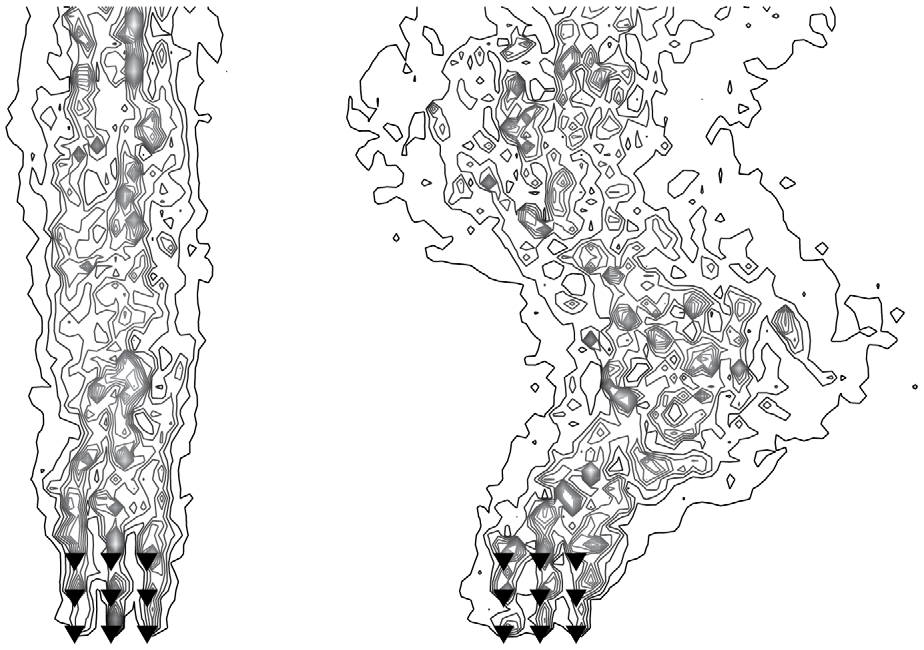
Examples of straight and meandering plumes with a superposed random velocity field. The triangles denote host position. The contours show concentration level, with darker bars indicating lower concentration. The lowest contour is 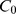 and is the same in both plots. The other contours are not the same in both plots because the maximum concentration is roughly three times higher in the meandering plume.

The odor plumes were produced by a regular arrangement of nine hosts with a density of 1 host per 1 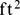 (0.09 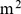) located in a single small patch in the center of the square simulation domain. The time series of random velocity fields superposed over the large-scale flow was the same for all simulations. See Table 1 for parameter choices and Eq. (2) for the formula for the meandering plume. An example of the general form of the meandering plume is shown in Fig. 5, alongside a straight plume for comparison. The meandering plume covers more area and achieves a greater width than the straight plume. The outermost contour in both plots is 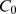, the 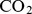 sensing threshold of the mosquito. The other contours are equally divided between 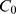 and the maximum concentration within each plume. The meandering plume has a maximum concentration that is approximately three times higher than that of the straight plume. Since the 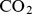 sources are the same in both simulations, the concentration difference is due solely to the differing velocity fields.

The results in Table 3 show the proportion of mosquitoes that found a host, 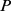, and the average time to locate a host, 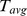. The column labeled 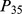 gives the value of 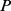 after only 35 s of host seeking. We make the following observations from the table.

- Given unlimited time, the proportion of mosquitoes that found a host was larger in the meandering plume for all plume-finding behaviors, but it took much longer on average to reach a host.
- In both plumes, a larger percentage of mosquitoes found a host using the crosswind strategy, but they took substantially longer to do so.
- If the mosquitoes were time-limited to 35 s, then results for upwind and downwind behaviors were essentially unchanged, but crosswind behavior went from being the most effective strategy to being the least effective in the meandering plume.

**Table 3:** A comparison of plume-finding behaviors in straight and meandering plumes.

plume-finding behavior	plume type	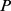	std dev	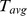	std dev	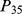
upwind	straight	22%	1.7%	11.6	0.5	22%
upwind	meander	38%	2.5%	15.9	0.4	38%
downwind	straight	20%	1.8%	4.6	0.9	20%
downwind	meander	39%	1.9%	9.7	0.6	38%
crosswind	straight	35%	3.5%	28.5	1.4	27%
crosswind	meander	57%	4.2%	56.9	2.0	14%

The third and fourth columns, 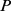 and std dev, are the average and standard deviation of the proportion of mosquitoes finding a host taken over 15 simulations of the same plumes with stochastic mosquito behavior. Each simulation is sufficiently long to ensure that all the mosquitoes either find a host or leave the domain. 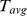 is the average time to a host taken over all simulations. The associated standard deviation taken over the *means* of the simulations. The final column recalculates 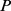 assuming that the simulation halts after 35 s of host-seeking.

### Assessing host distribution

We explored the number of contacts that occur in two host groups of equal density but unequal number. An equal per capita contact rate across both host groups would indicate that distributing the hosts into two separate patches has no effect. However we found unequal per capita rates between groups, indicating that a mosquito is less likely to detect a large group of hosts than it is to detect the same number of hosts distributed in smaller groups or as individuals. We also found unequal numbers of contacts between two unequally-sized groups, indicating that one group was easier to find than the other.

We performed a set of simulations in which we held the total number of hosts constant in the domain (10 birds), but split them between two groups in pairs of 9 and 1, 8 and 2, etc., down to 5 and 5 hosts per group. Fig. 2 shows an example of the 7-3 distribution. All other parameters were the same as in the previous section for the straight plume. The two straight plumes from the host groups were well separated from each other and from the left and right domain edges. The exact positions of the hosts were allowed to vary from one simulation to the next, and this affected the plume shape and the internal distribution of 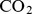 over the plume.

**Figure 6:**
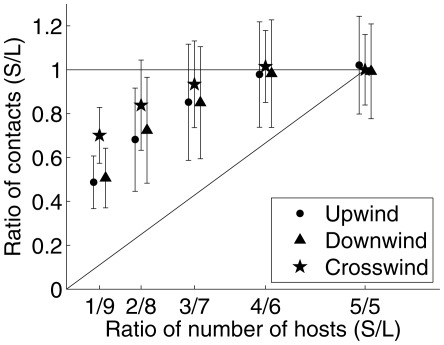
Ratio of contacts between a small and a large host group. Mean and standard deviation of the ratio of contacts in the small group to that in the large group (S/L) vs the ratio of group sizes. The line 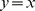 denotes an equal per capita contact rate between groups, while the line 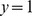 denotes an equal number of contacts at each group. The data corresponding to a given ratio of hosts have been separated slightly in the figure for visual purposes only.

To compare results between groups we plotted the ratio of the mosquito-host contacts in the smaller group divided by the mosquito-host contacts in the larger group (S/L) against the ratio of the number of hosts in the smaller group over the larger group (see Fig. 6). Each point in the error bar plot shows the mean and standard deviation over 150 simulations for each S/L ratio. The diagonal line in Fig. 6 denotes the case when the per capita contact rates are the same between groups. A value of 1 on the 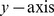 represents the case when the number of contacts was the same between both groups. When there are 5 hosts in both groups the number of contacts is the same, confirming that there is no left-right bias in the velocity field. When the group sizes are unequal, the per capita contact rates are higher in the small group, but the total number of contacts is higher in the large group (except for the nearly equal 6-4 distribution). This occurs in the region between the lines 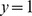 and 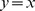. The crosswind strategy is closest to having equal numbers of contacts in both host groups, which corresponds to equal ease in locating either group.

### Assessing changing host density

In this section, we consider the effect of varying host density given a constant number of hosts. Intuitively, the size of the region where the hosts are congregated will affect the number of mosquito-host contacts for two reasons. First, a larger host area is more likely to be found by mosquitoes; second, the spatial arrangement of the hosts affects the size and shape of the odor plume they generate. We performed a set of simulations in which 10 hosts were distributed in a single patch in the center of the domain. Patch area was varied from 10–80 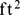 (1–7.4 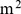) with a corresponding host density varying from 1–8 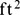 per host (0.1–0.74 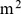). Our 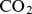 simulation region was 1076 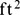 (100 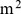), so that the patch occupied less than 10% of the simulation region. For each host density, we ran 150–450 simulations to average the effects of host position and individual mosquito choices, with more simulations performed for larger patch areas.

**Figure 7:**
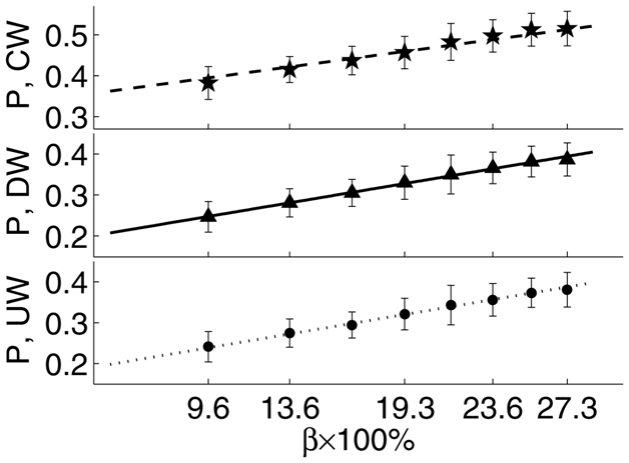
The proportion of mosquitoes finding a host as a function of host density. The simulation results are given by the solid markers with 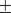 one standard deviation (UW = upwind, DW = downwind, CW = crosswind). The lines are linear fits to the data using the formula 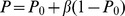, where 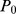 is a free parameter and 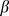 is the length of a side of the square host patch divided by the length of a side of the simulation region. As 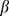 increases, the hosts spread out (i.e., decrease in density) and the number of mosquitoes locating a host increases. The 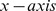 is 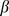 labeled as a percentage.

The proportion of mosquitoes 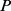 that made contact in the group is shown by the solid markers in Fig. 7, with the bars corresponding to 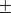 one standard deviation. All three plume-finding behaviors exhibit a slight upward trend in 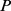 with increasing host patch area. Crosswind plume finding resulted in the most contacts, while upwind and downwind plume finding exhibit nearly identical results. In Fig. 7, 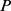 is plotted against the percentage 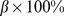, where 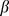 is the side length of the square host patch divided by the square computational domain side length. 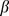 is a dimensionless ratio that relates a length scale important to the host distribution to one that is important to the mosquito distribution. As 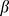 increases, the hosts are spread over a larger area and the host density decreases.

We approximated the results by the line 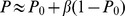, where 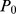 is the proportion of contacts made in the hypothetical case where the area of the host patch is zero. 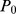 depends on plume-finding behavior, wind velocity, number of hosts, and other simulation parameters. We performed a least squares fit to find 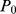 for each plume-finding behavior (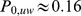, 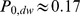, and 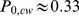) and the lines are shown in Fig. 7. In the Discussion, we present a first attempt to link this linear approximation from our agent-based model to a contact rate that has been used in standard (nonspatial) epidemiology models.

## Discussion

We developed an agent-based/continuum model to explore the effect of behavioral decisions and spatial heterogeneity on the contact rate between mosquito vectors and bird hosts and used it to address three issues of potential interest to researchers in epidemiology and vector control.

### Assessing plume-finding behavior

Our results generally show that crosswind plume finding most reliably led mosquitoes to a blood meal source. The effectiveness of the crosswind strategy over periods of minutes is compatible with the conclusions of the mark-release-recapture studies of *Anopheles gambiae* Giles in [34], where the dispersion of recaptured mosquitoes was related primarily to the distribution of human settlements (over time scales of days). We find that this effectiveness was accompanied by a high cost in time, as also seen in [12]. If the success of host location was restricted to occur within 35 s of mosquito release, then crosswind flight was the superior strategy only in a straight odor plume. In a meandering plume under the time limit, up- and downwind searching were better than crosswind flight. These results are similar to the conclusions drawn by Sabelis and Schippers [20], who used a geometric argument to show that crosswind plume finding is less effective when wind direction is highly variable.

### Assessing host distribution

When hosts were arranged in two groups of different size, the larger group consistently attracted more mosquitoes regardless of the plume-finding strategy, although the difference was less pronounced for crosswind flight (Fig. 6). At the same time, the smaller group experienced a higher per capita contact rate. These results are consistent with Foppa et al. [7], which reports on indoor experiments of *Cx. quinquefasciatus* feeding on roosting chickens. They found that the per capita feeding rate on a single chicken was about 4.27 times higher than that on a chicken in a group of nine. We find similar mean per capita ratios (4.3 and 4.4) for the upwind and downwind plume-finding behaviors when ten hosts are split into a group of nine and a singleton. The close match is surprising since our simulations included wind, whereas the experiments were conducted indoors. However, the uncertainty in the simulations and experiments is high.

The results indicate that it is advantageous for birds to roost together in larger groups on this spatial scale, because on average they will receive fewer bites. This phenomenon of attack abatement is well-known in the literature on predator-prey interactions [35], and likely has two causes in the situation considered here. First, the odor plume exuded by a group does not grow linearly with the number of hosts, because the hosts are clustered together. This leads to fewer mosquitoes locating the group than would find the same number of well-spaced individuals (an avoidance effect). Secondly, mosquitoes only need a fixed amount of blood, and so they will not attack additional individuals even if they are available. This is a dilution effect, also seen in the reduction of groups at risk for malaria resulting from urbanization [36]. The resulting contact heterogeneity arising from attack abatement could have important implications for transmission dynamics.

### Assessing changing host density

Standard models of mosquito-borne transmission assume that the mosquito contact rate on one host is inversely proportional to the numbers of hosts (reviewed, e.g. by [37]). This is likely true when hosts are so abundant that a mosquito will always be able to locate one. Our simulations indicate that the probability that a mosquito will locate a host is largely determined by the shape of the odor plume (see Table 3). However, the shape of an odor plume is difficult to predict, since it depends on local wind velocity and turbulence due to landscape features. We therefore propose an approximation that does not depend on plume shape, and is instead based on 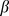, which can be viewed as a patchiness parameter (Fig. 7). This allows us to model contact rates when hosts are variably distributed over patches within a larger domain more realistically even if exact features of the relevant odor plumes are unknown.

We derive a patchiness-driven contact rate model by starting with the contact rate for malaria transmission from Chitnis et al. [38] which applies specifically in the case of homogeneous mixing in a fixed area without considering host-seeking mechanisms. They propose a vector-host contact rate of
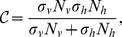
where 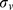 is the number of contacts each mosquito wants per unit time, 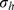 is the maximum number of contacts a host can receive per unit time, 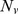 is the total number of vectors and 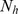 is the total number of hosts. This implies that 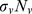 is the approximate contact rate when the mosquito population is small since every mosquito in a small population is expected to locate a host when the populations are homogeneously mixed.

We seek to modify this expression to account for host patch area, as depicted in Fig. 1, and mosquito behavior. When the hosts are limited to a small patch of area within a larger region, we make two modifications to the formula above. First, not every mosquito in a small population can be expected to find a host, since not all of the mosquitoes will come into contact with the odor plume. For this reason, we replace the contact rate with 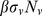, where 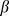 represents the ratio of the diameter of the patch where the hosts are located to the diameter of the 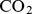 simulation region. The second modification comes from the fact that even when the patch size becomes small (and 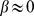), the number of contacts must be nonzero since even a tiny area produces a plume that mosquitoes can follow to a host. We want our modified contact rate function to be consistent with the original function in [38] when 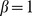, which is the case where the host patch is equal to the entire 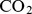 simulation region. Based on these observations we propose the modified contact function
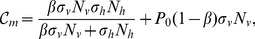
(8)where 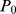 represents the proportion of mosquitoes that find a host as the patch of area containing the hosts becomes infinitesimally small.

We note that when the host patches are small (i.e. small 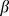), Eq. (8) can be linearized to read 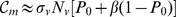, where the factor in brackets, 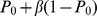, is interpreted as the proportion of mosquitoes that find a host for a given patch-to-region length ratio 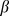. This proportion can be computed from the simulations, as shown by the lines in Fig. 7. Therefore, reasonable assumptions about the patchy distribution of hosts in the domain allow us to devise a quantitatively reasonable estimate of the host-finding probability of mosquitoes for the space and time scales considered here.

### Limitations

The length and time scales in the simulations presented here were motivated by the indoor experiments in [7] and a desire to quantify mosquito success near the end of a host-seeking flight. Our conclusions must be validated for spatial domains or time periods that are significantly larger. Furthermore, there are other probability distributions that can be used for mosquito navigational choices (see e.g. [12]) and a variety of initial mosquito spatial arrangements that may affect our conclusions.

**Figure 8:**
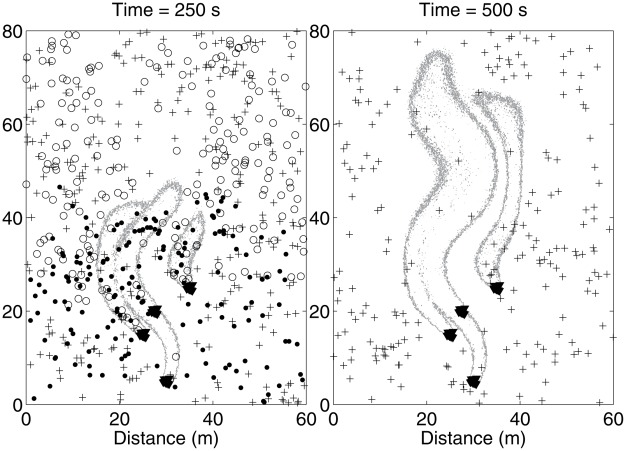
Growing odor plume in a large domain. Mosquitoes of all three plume-finding behaviors ( upwind, 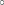 downwind, 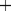 crosswind) near an evolving plume about 45–70 m long. All distances are in meters and only the outermost contour of the plume is shown. Left: The plume after 250 s. The upwind and downwind mosquitoes are already segregating into the upwind and downwind sides of the domain. Right: The same part of the domain after 500 s. The plume is about 2/3 longer and only crosswind mosquitoes remain in the vicinity of the plume.

Such parameter choices are within the capabilities of the model, and to demonstrate we briefly present a simulation of a growing odor plume in a large domain. Mosquitoes engaging in all three types of plume finding (2000 mosquitoes each) were initially uniformly distributed over a disk of radius 100 m centered on four clusters of ten roosting birds each. The wind meandered according to an incompressible flow appropriate for the large domain size and the crosswind mosquitoes flew in the same direction for a number of decisions chosen from a Lévy distribution having a power law in the tail of 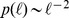 [39]. The Lévy distribution was centered at 0.7 s, the mean value of 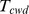 in Table 1, and only positive values were sampled. The mosquitoes were placed in the domain after 150 seconds when the odor plume was approximately 35 meters long. Fig. 8 shows the state of the odor plume after 250 s and 500 s, along with the positions of all nearby mosquitoes. The simulation was run for 1500 s, at which point the odor plume was about 180 m in length and 4.2%, 6.4%, and 26.6% of the upwind, downwind, and crosswind mosquitoes had located a host respectively. The success rate was lower compared to the smaller domain, particularly for the upwind and downwind mosquitoes that rapidly left the plume behind (see Fig. 8, right). The crosswind mosquitoes moved downwind at the same rate the plume did, and therefore had many more opportunities to intercept it. There were still a substantial number of crosswind mosquitoes interacting with the plume when the simulation ceased.

We hope to expand our careful analysis of the smaller domain to a larger domain in the future. In larger domains, it would be interesting to include the effect of mosquito breeding sites and how their locations affect the biting rate of hosts near them in comparison to hosts living farther away. Significant differences in the biting rates in spatial relation to breeding sites have been reported for malaria in [40].

### Future directions

There are other potential model extensions that are equally interesting. Disease transmission via mosquito bite, host movement, infection, and demographic processes in both vertebrate hosts and mosquitoes and the dependence of these processes on biotic and abiotic factors could be integrated with the existing model for explicit small-scale modeling of disease spread. This modeling framework is capable of accommodating many further levels of complexity, such as gusting wind, moving hosts, multiple host types, odor-baited traps, variable breathing rate, compound odors, repellents, etc. The challenge will be to identify the components that most strongly affect the behavior of the model system and the underlying reality on which it is based. For example, in the simulations presented here the spread of the host odor is dominated by turbulent convection and diffusion plays a very minor role. It is therefore reasonable to hypothesize that the particular odor cue used by the mosquitoes will have little effect, and that substituting lactic acid for 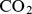 (for example) will not impact the resultant contact rates. See [13] for a discussion of turbulent mixing versus molecular diffusion in the context of chemotaxis.

Additional factors could be included in the model in order to capture subtle differences between mosquito species, lighting effects, and other elements not included in the current work. Hosts tend to attract mosquitoes in unequal ways [41]. Differential attractiveness, the emission of different levels of 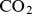 by different hosts as well as multiple odor cues can also be introduced into the model to study situations like those presented in [42], where mosquitoes of many species finding humans distributed in huts, or in [43], where mosquitoes were collected inside village houses in Tanzania. These studies report an approximate direct relationship between the number of inhabitants per house and the number of mosquitoes collected. Our model could be used to study which factors influence more strongly this relationship.

From the point of view of controlling the vector population, the model presented here may offer some insights into how the spatial distribution of mosquito traps may affect the overall control. This can be accomplished by replacing the hosts in the model with odor-baited mosquito traps and adjusting appropriate parameters. This was addressed in a non-spatial model by Okumu et al. [10], where homogeneous mixing of hosts was assumed. They discuss the importance of space in the vector-host contact process and indicate that the rate at which an individual host is discovered by an individual vector depends on the distance between hosts and vectors as well as on the size of the odor plumes generated by the hosts. Further, spatial characteristics such as the topography and wind direction are known to be influential in the rates at which individual hosts are found. Our model can explicitly include spatial features to compare strategies of where to place mosquito traps relative to blood-source hosts. According to the studies in [44] the distances at which various species of mosquitoes responded to 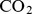 baits by initiating orientation toward the them was 30 meters or less. Therefore, the small model length scales presented here are appropriate for such simulations.

## References

[1] Woolhouse ME, Dye C, Etard JF, Smith T, Charlwood JD (1997). Heterogeneities in the transmission of infectious agents: Implications for the design of control programs.. PNAS.

[2] Hasibeder G, Dye C (1988). Population-dynamics of mosquito-borne disease - persistence in a completely heterogeneous environment.. Theor Popul Biol.

[3] Smith DL, Dushoff J, McKenzie FE (2004). The risk of a mosquito-borne infection in a heterogeneous environment.. PLoS Biol.

[4] Smith DL, Dushoff J, Snow RW, Hay SI (2005). The entomological inoculation rate and plasmodium falciparum infection in African children.. Nature.

[5] Dye C, Hasibeder G (1986). Population-dynamics of mosquito-borne disease - effects of ies which bite some people more frequently than others.. Trans R Soc Trop Med Hyg.

[6] Lehane MJ (1991). Biology of blood-sucking insects.

[7] Foppa IM, Moore J, Caillouët KA, Wesson DM (2011). Disproportionate mosquito feeding on aggregated hosts.. J Med Entomol.

[8] Killeen GF, Smith TA, Ferguson HM, Mshinda H, Abdulla S (2007). Preventing childhood malaria in Africa by protecting adults from mosquitoes with insecticide-treated nets.. PLoS Med.

[9] Killeen GF, Smith TA (2007). Exploring the contributions of bed nets, cattle, insecticides and excitorepellency to malaria control: A deterministic model of mosquito host-seeking behaviour and mortality.. Trans R Soc Trop Med Hyg.

[10] Okumu FO, Govella NJ, Moore SJ, Chitnis N, Killeen GF (2010). Potential benefits, limitations and target product-profiles of odor-baited mosquito traps for malaria control in Africa.. PloS ONE.

[11] Le Menach A, Takala S, McKenzie FE, Perisse A, Harris A (2007). An elaborated feeding cycle model for reductions in vectorial capacity of night-biting mosquitoes by insecticide-treated nets.. Malaria.

[12] Pasternak Z, Bartumeus F, Grasso FW (2009). Lévy-taxis: A novel search strategy for finding odor plumes in turbulent ow-dominated environments.. J Phys A: Math Theor.

[13] Vickers NJ (2000). Mechanisms of animal navigation in odor plumes.. Biol Bull.

[14] Bidlingmayer WL (1994). How mosquitoes see traps: role of visual responses.. J Am Mosq Control Assoc.

[15] Clements AN (1999). Biology of Mosquitoes. Volume 2, Sensory Reception and Behaviour.

[16] Dekker T, Geier M, Carde RT (2005). Carbon dioxide instantly sensitizes female yellow fever mosquitoes to human skin odours.. J Exp Biol.

[17] Gibson G, Torr SJ (1999). Visual and olfactory responses of haematophagous Diptera to host stimuli.. Med Vet Entomol.

[18] Gillies MT, Wilkes TJ (1974). Evidence for downwind ights by host-seeking mosquitoes.. Nature.

[19] Dusenbery DB (1989). Optimal search direction for an animal ying or swimming in a wind or current.. J Chem Ecol.

[20] Sabelis MW, Schippers P (1984). Variable wind directions and anemotactic strategies of searching for an odour plume.. Oecologia.

[21] Service MW (1980). Effects of wind on the behaviour and distribution of mosquitoes and blackies.. Int J Biometeorol.

[22] Bowen MF (1991). The sensory physiology of host-seeking behavior in mosquitoes.. Annu Rev Entomol.

[23] Gillies MT (1980). The role of carbon dioxide in host-finding by mosquitoes (Diptera: Culicidae): A review.. Bull Entomol Res.

[24] Dekker T, Takken W, Carde RT (2001). Structure of host-odour plumes inuences catch of *Anopheles gambiae s.s*. and *Aedes aegypti* in a dual-choice olfactometer.. Physiol Entomol.

[25] Dekker T, Carde RT (2011). Moment-to-moment ight manoeuvres of the female yellow fever mosquito (*Aedes aegypti* L.) in response to plumes of carbon dioxide and human skin odour.. J Exp Biol.

[26] Syed Z, Leal WS (2009). Acute olfactory response of *Culex* mosquitoes to a human-and bird-derived attractant.. PNAS.

[27] Cardé RT (1996). Odour plumes and odour-mediate flight in insects.. Ciba Foundation Symposium, No. 200. Olfaction in mosquito-host interactions.

[28] Cooperband MF, Cardé RT (2006). Orientation of Culex mosquitoes to carbon dioxide-baited traps: flight manoeuvres and trapping efficiency.. Med Vet Entomol.

[29] Davis EE (1996). Olfactory control of mosquito behavior.. Ciba Foundation Symposium No. 200. Olfaction in mosquito-host interactions.

[30] LeVeque RJ (1996). High-resolution conservative algorithms for advection in incompressible flow.. SIAM J Numer Anal.

[31] Keller EF, Segel LA (1971). Model for chemotaxis.. J Theor Biol.

[32] Horstmann D (2003). From 1970 until present: The Keller-Segel model in chemotaxis and its consequences I.. Jahresbericht der DMV.

[33] Pierce-Shimomura JT, Morse TM, Lockery SR (1999). The fundamental role of pirouettes in *Caenorhabditis elegans* chemotaxis.. J Neurosci.

[34] Gillies MT (1961). Studies on the dispersion and survival of *Anopheles gambiae* Giles in East Africa, by means of marking and release experiments.. Bull Entomol Res.

[35] Turner GF, Pitcher TJ (1986). Attack abatement: A model for group protection by combined avoidance and dilution I.. Am Nat.

[36] Hay SI, Guerra CA, Tatem AJ, Atkinson PM, Snow RW (2005). Urbanization, malaria transmission and disease burden in Africa.. Nat Rev Microbiol.

[37] Wonham MJ, Lewis MA, Renclawowicz J, van den Driessche P (2006). Transmission assumptions generate conicting predictions in host-vector disease models: A case study in West Nile virus.. Ecol Lett.

[38] Chitnis N, Cushing JM, Hyman JM (2006). Bifurcation analysis of a mathematical model for malaria transmission.. SIAM J Appl Math.

[39] Reynolds AM, Frye MA (2007). Free-ight odor tracking in *Drosophila* is consistent with an optimal intermittent scale-free search.. PLoS ONE.

[40] Thompson R, Begtrup K, Cuamba N, Dgedge M, Mendis C (1997). The Matola malaria project: A temporal and spatial study of malaria transmission and disease in a suburban area of Maputo, Mozambique.. Am J Trop Med Hyg.

[41] Knols BG, de Jong R, Takken W (1995). Differential attractiveness of isolated humans to mosquitoes in Tanzania.. Trans R Soc Trop Med Hyg.

[42] Haddow AJ (1942). The mosquito fauna and climate of native huts at Kisumu, Kenya.. Bull Entomol Res.

[43] Killeen G, Tami A, Kihonda J, Okumu F, Kotas M (2007). Cost-sharing strategies combining targeted public subsidies with private-sector delivery achieve high bednet coverage and reduced malaria transmission in Kilombero Valley, southern Tanzania.. BMC Infect Dis.

[44] Gillies MT, Wilkes TJ (1972). The range of attraction of animal baits and carbon dioxide for mosquitoes. Studies in a freshwater area of West Africa.. Bull Entomol Res.

